# Using XRF and ICP-OES in Biosorption Studies

**DOI:** 10.3390/molecules23082076

**Published:** 2018-08-19

**Authors:** Katarzyna Chojnacka, Mateusz Samoraj, Łukasz Tuhy, Izabela Michalak, Małgorzata Mironiuk, Marcin Mikulewicz

**Affiliations:** 1 Department of Advanced Material Technologies, Faculty of Chemistry, Wrocław University of Science and Technology, Smoluchowskiego 25, 50-372 Wrocław, Poland; katarzyna.chojnacka@pwr.edu.pl (K.C.); Mateusz.Samoraj@grupaazoty.com (M.S.); Lukasz.Tuhy@grupaazoty.com (L.T.); izabela.michalak@pwr.edu.pl (I.M.); 2 Department of Dentofacial Orthopeadics and Orthodontics, Division of Facial Abnormalities, Medical University of Wrocław, ul. Krakowska 25, 50-425Wrocław, Poland; mikulewicz.marcin@gmail.com

**Keywords:** multielemental analysis, ICP-OES, XRF, biomass, biosorption, surface concentration

## Abstract

In this work, a method of recalculation of results of X-ray fluorescence (XRF) technique to Inductively Coupled Plasma-Optical Emission Spectroscopy (ICP-OES) method was elaborated for biosorption studies. Equations that calibrate XRF to ICP-OES were determined, as a biosorbent strawberry, blackcurrant and raspberry seeds after supercritical CO_2_ extraction were used. ICP-OES showed a better precision and lower detection limits than XRF. The latter technique is cheaper, requires minimal sample preparation and gives faster results. Linear regression of the data gave almost 1:1 correlations without additional correction (for Cu r^2^ = 0.9998, Mn r^2^ = 0.807, Zn r^2^ = 0.979). Calibration and quantification of intensities of XRF was obtained using ICP-OES measurements after samples digestion with HNO_3_ in a microwave system. High positive correlations were estimated for Cu, Mn, Zn. It was demonstrated that XRF technique can be used together with other well established techniques (ICP-OES) to produce quantitative data from biosorption studies. Elaboration of cheap and quick analytical methodology is an important aspect in development of new processes and products based on biosorption process.

## 1. Introduction

Developing sustainable analytical methods can significantly reduce the use of concentrated mineral acids (e.g., nitric acid), that are required for samples digestion, energy required to operate analytical instruments (e.g., plasma spectrometers) or technical gases. This is the area of GAC (Green Analytical Chemistry). It is an important challenge because the use of this approach reduces the use of non-environmentally friendly chemicals and energy. In this context, finding green analytical methods to replace non-green is an important target which fits into the topic of green chemistry and green analytical chemistry [1].

By using simple and direct methods of chemical analysis, environmental side effects of chemical activities of analytical process should be taken into consideration. Green Analytical Chemistry concerns all aspects of the analysis of any kinds of sample, not only those for environmental studies. There are 12 principles of GAC (Table 1). Using cheap, fast and environmentally friendly analytical methods is the challenge to increase demand for low-chemicals and low-energy consuming and cheap methodologies. 

It is necessary to take into consideration that green analytical methods might have lower sensitivity or even have semi-quantitative character. GAC lowers the reduction of the waste generated, eliminates toxic reagents and assures softer conditions, reduces of energy consumption, time, reagents and waste, miniaturization, preference for reagents obtained from renewable source, efficient energy use and enables minimization of risk of accidents [3]. 

The idea of green chemistry has origins in sustainable development. According to GAC, the role of analytical chemists in making laboratory procedures more environmentally friendly, thereby efforts are being made to reduce the negative impact of chemical analyses on the environment. A very important challenge is to reach a compromise between the quality of the results and the environmental friendliness of analytical methods [2]. 

In many analytical methods, samples composition is determined after extraction from the solid matrices. If the sample is of biological origin, it is composed of sugars, proteins, lipids, fibers and other organic and mineral constituents. The introduction of sample-preparation methods is a necessary step of chemical analysis. The main problem of conventional sample-preparation methods arises from high consumption of mineral acids required for samples mineralization, that is cost, time and energy-consuming. These shortcomings have led to consideration of methods of direct analysis, the results of which are verified by conventional non-green, but sensitive methods [4].

In the present work, XRF (X-Ray Fluorescence) method was used as a green analytical method to determine the composition of micronutrients bound by the bio-based materials in the process of biosorption. Biosorption is defined as the process of molecules binding from aqueous solution to non-living biomass, related with binding of metal ions from aqueous solution to chemical groups present on the surface of the cell wall of the biomass. Biosorption is a method of contaminants removal from effluents, but also the method of production of micronutrient feed additives and fertilizer components. Since the carrier of micronutrients is of biological origin, the quality of the product and its standardization should be frequently controlled. This causes the need to develop a fast, cheap and eco-friendly method of analysis of these bio-based products. Because the product itself is eco-friendly, analytical methods should follow the same trend. Since the density of micronutrients on the surface of biosorbent material is high, the methods of analysis do not necessarily are required to have high limit of detection. The idea of the present work is to use cheap and fast method of analysis (XRF) for quality control of process and products. The same samples were analysed quantitatively by non-ecofriendly ICP-OES method (Inductively Coupled Plasma Optical Emission Spectrometry). The results obtained by both methods were correlated to make it possible to recalculate the results from XRF to ICP-OES results.

Mahmoud and Fawzy described biosorbents as materials for the removal of toxic metal ions from the solutions. In order to determine environmental factors that influence biosorption isotherm, equilibrium, and kinetic models several analytical determinations need to be carried out [5]. In order to successfully apply the process of biosorption in practice, simple, cheap and reliable analytical methods are required [6]. Efficient removal of undesirable metals from wastewater is still challenging, not only from the view of technology itself, but also as a task for the proper selection of analytical techniques. The physicochemical characteristics of the surfaces of biosorbents determines the selection of analytical methods, as the sensitivity and the selectivity of these materials are related with their surface. In this context, the possibility of surface analysis is important in applicability of analytical methods. [7].

With increasing interest in biosorption, the development of the cost-effective and reliable methods of quantitative description of the process is required. The most commonly used analytical tools are based on the analysis of the solubilized biomass AAS (Atomic Absorption Spectroscopy), ICP-MS (Inductively Coupled Plasma Mass Spectrometry), ICPOES or direct methods of analysis: SEM-EDX (Scanning Electron Microscopy with Energy Dispersive X-Ray Spectroscopy), TEM (Transmission Electron Microscopy) that enable to determine the density of elements bound to the surface of biomass.

The determination of the composition of enriched biomass by ICP-OES involves sample mineralization and is time-consuming. Previously, we correlated the results obtained by ICP-OES with other direct measurement technique: SEM-EDX [8,9]. A number of articles, showing the application of ICP–OES in quantitative description of biosorption process, proved high efficiency of this method in investigation of biosorption phenomenon [9]. The method was widely used in biosorption studies independently from further application of enriched biomass (wastewater treatment, feed additives, fertilizer components). ICP-OES was shown to be useful in the determination of toxic metal and micronutrient ions. The analysis of elemental composition of enriched biomass requires mineralization with the use of strong acids in microwave system what makes the method destructive but enables to determine the content of metal ions in the whole volume of the sorbent—not only on its surface [8]. 

Among the methods of determination of element content in different materials, XRF (X-ray fluorescence) was shown to be widely used. There are many reports describing the application of XRF in the analysis of the elemental composition of solid samples characterized by high accuracy of measurement. X-ray fluorescence constitutes a convenient tool for the analysis of major elements in solid samples [10]. Fittschen and Falkenberg [11] pointed out that XRF can be useful in the environmental analysis for the evaluation of the presence of metal ions in different materials (soil and particles, plants, vertebrates and invertebrates). XRF was shown to be an effective tool for the determination of the element content on the surface of different materials: bones [12], soil [13,14], and plants [15]. This method is quick, cheap and no-destructive towards biological material. Furthermore, the analysis can be made with the use of portable device and there is no necessity for the additional interpretation of obtained results as it is in the case of ICP-OES. However, data about the application of XRF in biosorption process (determination of metal content in the biomass) are scarce. This method was also used for analysis of biosorption of Fe(II) and Mn(II) ions from aqueous solution by rice husk ash [16], for Cd(II) biosorption by macrofungal biomass (*Clitopilus scyphoides*) [17] and for Pb(II) biosorption by termite mound [18].

**Figure 1:**
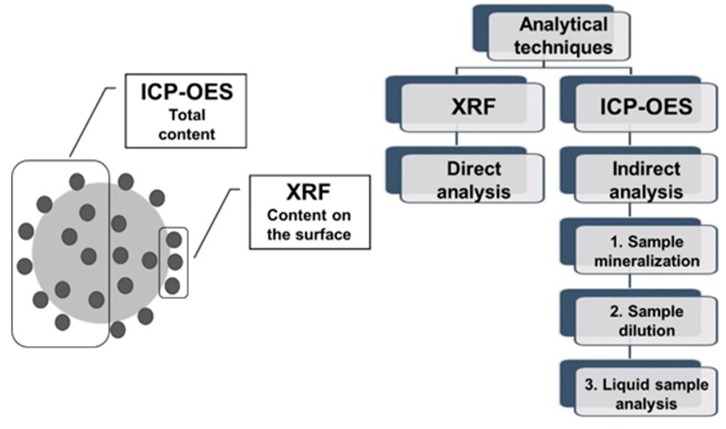
Comparison of ICP-OES and XRF in biosorption studies.

The cross-correlations of X-ray fluorescence results with ICP-OES were described in the literature, for example for sediment samples [19], soil samples [20,21,22], municipal landfill leachate [23], dental ceramics [10], air filter samples [24]. Figure 1 illustrates differences between described analytical methods.

**Figure 2:**
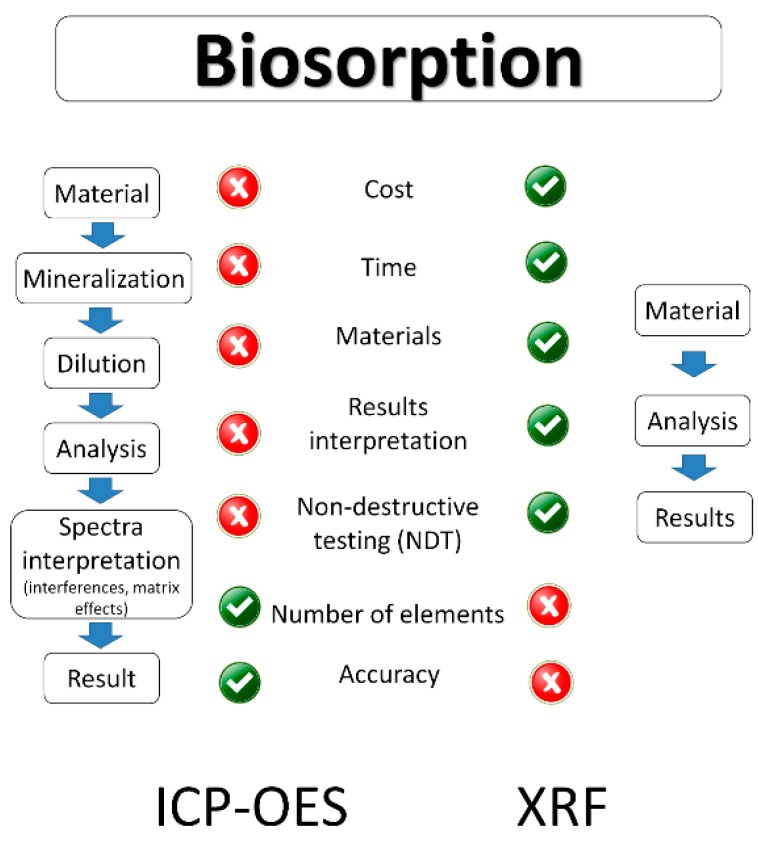
Advantages and disadvantages of ICP-OES and XRF in biosorption studies.

The analysis of samples by ICP-OES preceded by the acid mineralization is time consuming and expensive when compared to portable X-ray fluorescence [14]. The comparison of both techniques is presented in Figure 2.

There are over 13,000 papers published on the topic of biosorption, only in the last three years: 4100 papers have been published (source: ISI Web of Science). The majority of those papers include research, whereby advanced instrumental analytical techniques have been used, e.g., AAS, ICP-OES or ICP-MS. Since biosorption is a process associated only with the surface of the material, and methods such as AAS, ICP-OES, ICP-MS measure the content in the whole mass of the sample. Methods dedicated to the determination of the surface composition, e.g., XRF could be more justified. The methods, such as ICP-OES/MS are reliable and characterized with high precision and sensitivity, however cannot be considered as green analytical methods. A huge amount of experimental work requires numerous analytical determinations. Green analytical methods (e.g., XRF) are semi-quantitative methods that require validation and standardization for specific applications [25].

The paper reports results on elaboration of green analytical methods designated for determination of microelements in biosorption studies. Recently, new applications of biosorption process have been developed, addressed to produce new fertilizers and dietary feed supplements with microelements. The elaboration of new technologies should be accompanied by with the development of new analytical methods that will be used to control the quality of processes and products. Analytical methods used routinely in industry should be cheap, simple and quick. Therefore, they fit into the requirements set for green analytical methods.

The aim of the present work was to examine and compare by cross-calibration two analytical techniques—ICP-OES and XRF in quantitative description of the biosorption of Zn(II), Mn(II) and Cu(II) ions by post-extraction residues of berries seeds (strawberry, blackcurrant, raspberry). Berries seeds of these fruits were used for the production of essential oils in the supercritical fluid extraction process. The obtained residue requires utilization. Biosorption process of microelement ions allows to obtain natural valuable components of fertilizers.

## 2. Results

Comparison of results obtained from the analysis of the post-extraction residues enriched with Zn, Mn and Cu ions by biosorption by ICP-OES and XRF is presented in Table 2. The materials were obtained as post-extraction residues from Supercritical Fluid Extraction Process. The materials then underwent biosorption process to enrich with Cu, Mn and Zn ions.

In the present paper, in order to compare the content of a given microelement in the biomass, enrichment coefficient was introduced (1):

(1) $$\alpha =\frac{C_{E}}{C_{B}}$$

where: *C_B_* content of microelement in raw biomass (mg/kg), *C_E_* content of microelement in enriched biomass (mg/kg), *α* enrichment coefficient. This coefficient was determined for both methods. The results are presented in Table 3.

In Table 4, the coefficients (XRF/ICP-OES) for the content of Zn, Cu and Mn in natural and enriched biomass were compared. It was shown that XRF/ICP-OES coefficients values were, in most cases, higher in enriched biomass.

**Figure 3:**
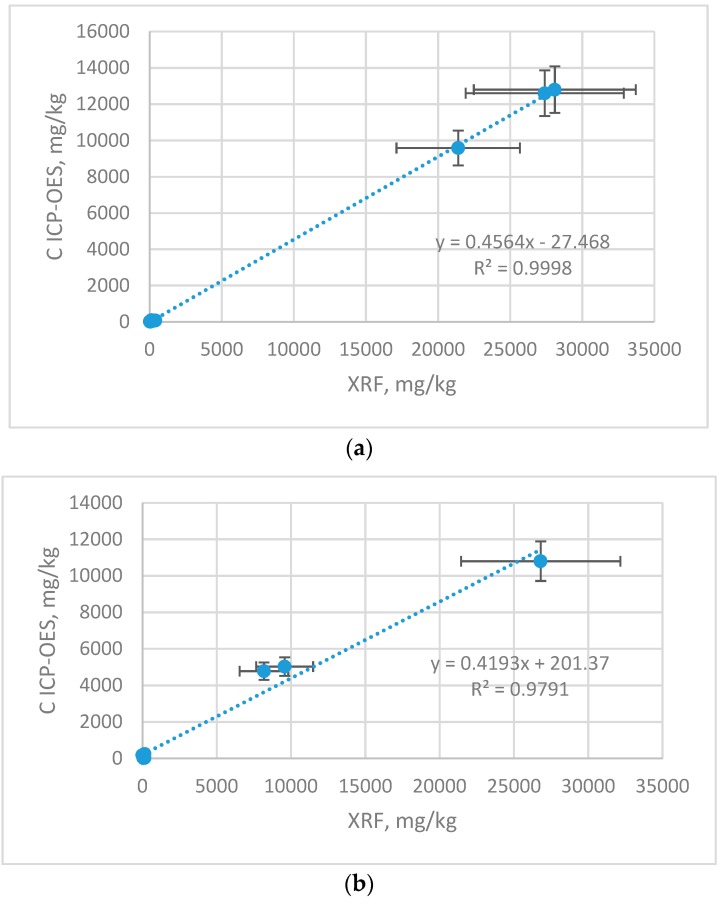

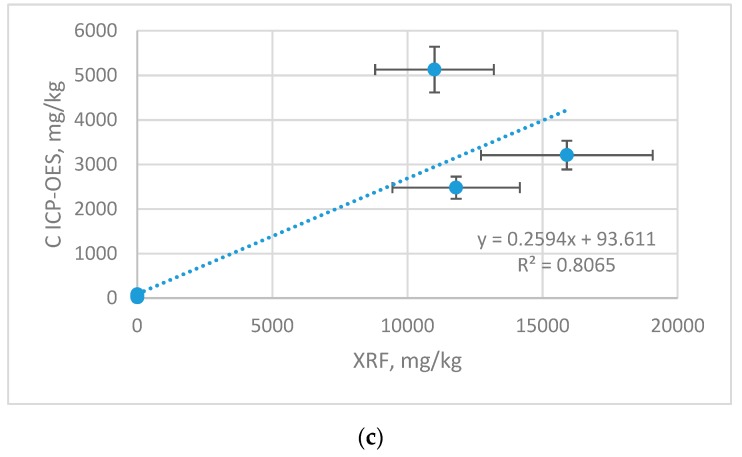
Correlation between XRF and ICP-OES results (**a**) Cu; (**b**) Zn; (**c**) Mn.

**Figure 4:**
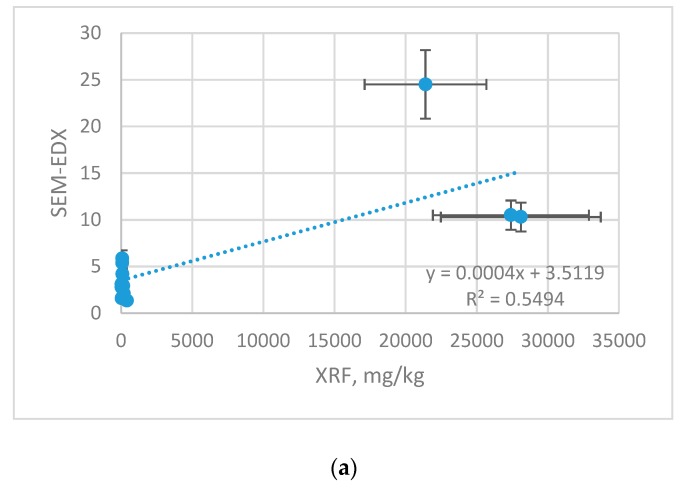

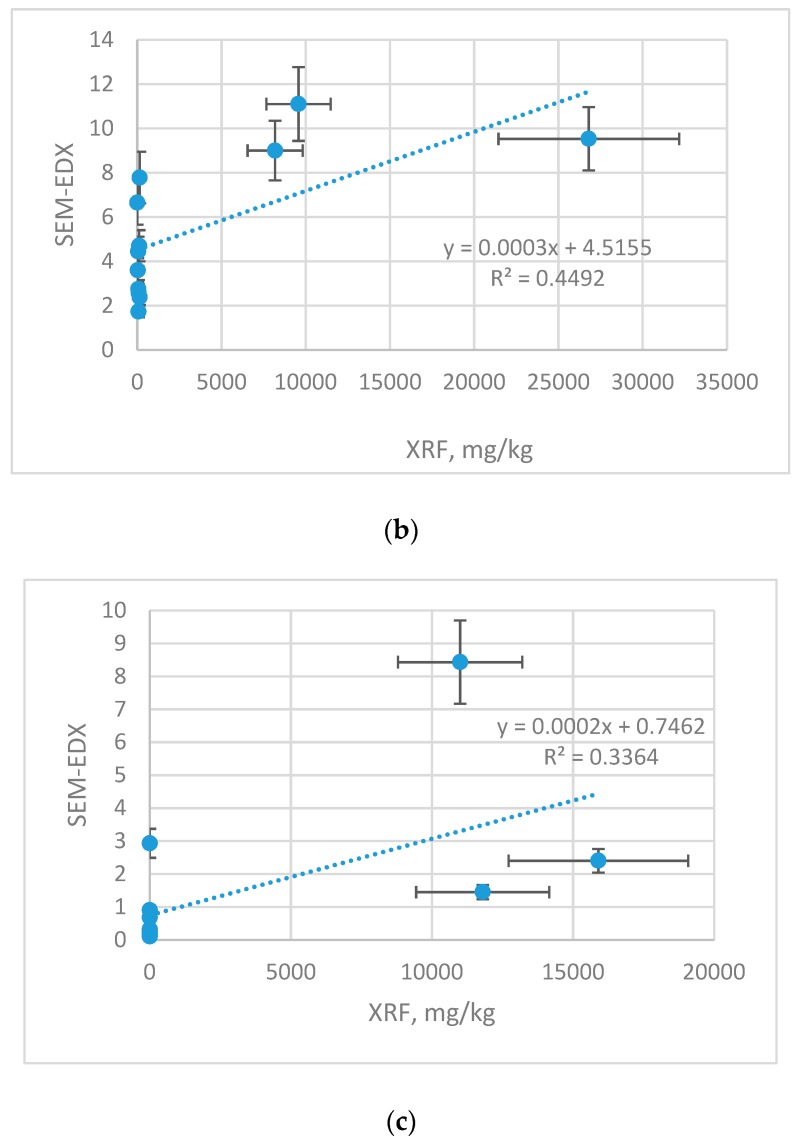
Correlation between XRF and SEM-EDX results [26]: (**a**) Cu; (**b**) Zn; (**c**) Mn.

Figure 3 and Figure 4 report biosoption capacity of the biosorbent for Cu, Mn and Zn ions. The value of biosorption capacity corresponded to the residual concentration of metal ions in the solution. In Figure 3 correlation graphs for Zn, Cu and Mn content in samples (ICP-OES) and on samples surface (XRF) are shown. The results are shown with the error bars, which represent with the uncertainty of the measurement.

There is a strong linear cross-correlation between the content determined by ICP-OES and XRF for Cu r 0.9999, Zn r 0.9895 and less strong for Mn r 0.8980. The higher content of given elements, the correlation coefficient comes close to 1. This is related to the detection limits of both methods. The ICP-OES technique has better sensitivity and lower detection limits compared to the XRF method, therefore using this method for determining lower content is burdened with higher error, and the correlation with ICP-OES is weaker. Cross-calibration makes it possible to recalculate the results from non-destructive, simple and cheap XRF method to ICP-OES to make it more meaningful. Table 5 reports equations that make this recalculation possible. Because biosorption is the process related with the sorbent surface, the level of analytes will be above LLD. 

XRF results were also related with other technique SEM-EDX (Figure 4). The results are shown with the error bars, which represent with the uncertainty of the measurement.

Although both techniques rely on identification of the surface composition, the cross-correlation yielded significantly lower agreement than XRF vs. ICP-OES technique. For XRF vs. SEM-EDX, the differences originate from diversified LLD and were evaluated as follows: Cu (r 0.741), Zn (r 0.670) and Mn (r 0.580). Table 6 reports equations for recalculation of XRF vs. SEM-EDX results. 

**Figure 5:**
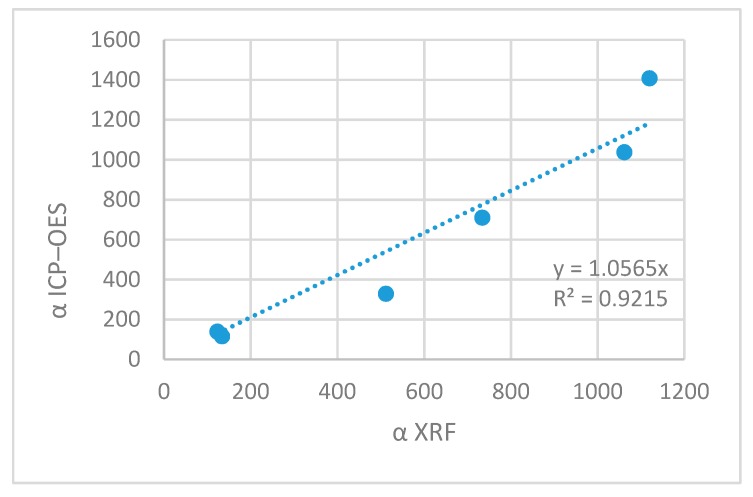
Enrichment coefficient determined by XRF vs. ICP-OES.

Low cross-correlation of results of both methods could be related with the fact that both techniques are of semi-quantitative character and correlating the results yields lower data agreement. Figure 5 reports enrichment coefficients determined for Zn and Cu by both methods: XRF and ICP-OES.

Enrichment coefficient determines how many times the content of micronutrients increased in the sorbent as the result of biosorption. This is the mean of final interpretation of biosorption process. The results stay in agreement very well. The cross-correlation was evaluated as r 0.9600 with the correlation equation y = 1.06x. This shows that the results obtained by both methods are comparable and yield coherent interpretation of the results. This shows that XRF is a simple and non-destructive method can be used to control the course of the biosorption process and the quality of products. This is an important issue in elaboration of chemical technology and accompanying analytical methods.

## 3. Discussion

Generally, values obtained for XRF method were higher than for ICP-OES in the examined samples (Table 2) for Zn, the content was averagely 2.5 ± 1.3 times higher and ranged from 1.6 to 5.3, for Cu was 4.0 ± 2.3 times higher and ranged from 1.6 to 7.8 and for Mn—4.0 ± 1.6 times higher and ranged from 2.1 to 5.0. 

According to Table 3, for all enriched post-extraction residues, the highest enrichment coefficient determined from ICP-OES data was obtained for Cu(II), then for Zn(II) and finally for Mn(II) ions. It was impossible to determine α for Mn(II) ions because the content in the raw biomass was below detection limit of this technique. There are visible differences in elements content between natural and micronutrient-loaded samples, in both, ICP-OES and XRF analyses, however, the enrichment coefficient for both methods is comparable (beside blackcurrant—1.5 times higher for XRF than for ICP-OES).

The highest enrichment coefficient obtained for Cu(II), then for Zn(II) and finally for Mn(II) (Table 4) can be explained by characteristics of the metal ions. Based on the data presented in the publication of Can and Jianlong [27], the relationship between ionic characteristics and the content of metal ions in the enriched biomass was examined. Taking into account several values: oxidation number (OX)—2 for all examined elements Cu(II), Zn(II), Mn(II); atomic weight (AW)—Cu: 63.5, Zn: 65.4, Mn: 54.9; atomic radius (AR)—Cu: 1.35, Zn: 1.31, Mn: 1.12; ionization potential (IP)—Cu: 20.3, Zn: 18.0, Mn: 15.6; electronegativity (Xm)—Cu: 1.9, Zn: 1.65, Mn: 1.55, it can be concluded that probably IP and Xm are responsible for the highest biosorption capacity of the examined biomasses for Cu(II) ions. 

The disparity between XRF and ICP-OES values was also observed by Arenas and coworkers [20], who examined geochemical characterization of the mining district. The content of Cu was 5.8 times higher for XRF than for ICP-OES, Zn—1.3 times higher and for Mn the values were comparable. In the other work [21], the content of Cu, Zn and Mn in soil samples determined by XRF were 2.4 times higher than the values determined by ICP-OES. This could be explained by the fact, that XRF measured the content of metals on the surface of a sample. In the case of ICP method, which requires samples solubilization by mineralization, the content is measured in the whole sample (outside and inside of the sample). XRF can be applied not only for solid samples, but also for the liquid. Cataldo [23] performed multielemental analysis of a municipal landfill leachate with TXRF (Total Reflection X-Ray Fluorescence) and with ICP-OES. The concentration of Cu was comparable for both techniques (ICP—0.030 and XRF 0.028 mg/L), for Mn and Zn was much higher for XRF (Mn: ICP—0.750 and XRF 0.944 mg/L; Zn: ICP—0.306 and XRF 0.538 mg/L). Despite the differences in the values of the element content in samples for both methods, the agreements between ICP-OES and XRF results exist and have high correlation coefficients. The highest determination coefficient between techniques was found for Cu (0.9998), but for other micronutrients this coefficient was also high Mn—0.807, Zn—0.979. The levels of elements obtained for X-ray fluorescence gave strong correlation with results obtained with the use of ICP-OES. 

These results are in agreement with literature data. Good correlation between ICP-OES and XRF was also proved by examination the content of Pb in solid samples (air filter samples from a lead ore concentrator mill and a lead-acid battery recycler) [24]. Correlations between XRF and ICP values close to 1:1 were obtained for most samplers. The contamination of soil samples with toxic metals (Cu, Pb, As, Cd, Zn, Fe, Ni and Mn) was examined, good agreement between results obtained by ICP-OES and XRF for trace elements was indicated [21]. Similar, results were presented in publication of Coetze and al., who compared these two methods in the study on determination of trace elements in soil and grass samples [28]. The good correlation between methods and similar precision were achieved. Opposite results were obtained in other studies on Pb and As level in soil samples using ICP-OES and XRF [24]. A significant difference in Pb levels measured with the use of these two analytical methods were reported, whereas no statistically significant differences in the level of As were observed.

Strong correlations between ICP-OES and XRF were shown also for liquid samples, where the multielement content of municipal landfill leachates was examined [26]. A straight line was obtained (for Hg, Pb, Cu, V, Ni, As, Cr, Zn, Ti, Mn, B, Rb, P, Sr, Fe, Br, Mg, S, Ca, K, Na, Cl) by putting in ordinate the analytical results of the ICP-OES and in abscissa the analytical results of the TXRF.

Conducted experiments showed that XRF can be used for quantitative description of biosorption phenomenon despite the fact that it enables examination of element content of the surface of biomass, not in the whole volume of probe. It was also observed that the range of elements which could be examined by XRF was not as wide as for ICP-OES.

## 4. Materials and Methods

For the experiments, post-extraction residues of strawberry, blackcurrant and raspberry seeds after supercritical CO_2_ extraction, delivered by New Chemical Syntheses Institute (Puławy, Poland) were used. The biosorption of Zn(II), Cu(II) and Mn(II) ions was conducted in batch mode in stirred tank reactors (60 L). The concentration of zinc (ZnSO_4_∙7H_2_O, POCH, Poland), copper (CuSO_4_∙5H_2_O, POCH, Poland) and manganese (MnSO_4_∙H_2_O, POCH, Poland) in the solution was about 300 mg/L for each process, pH was measured in 25 °C with the use of pH meter Mettler Toledo SevenMulti, Switzerland and was adjusted to 5. The concentration of the biomass was 1.0 g of dry mass (d.m.)/L. In each process, 40 g of biosorbent was used. The mixture was stirred by aerating with the air pump for an hour. The solution was filtered and the biomass was dried at 50 °C for 24 h. The content of elements in the enriched biomass was examined by XRF and ICP-OES.

### 4.1. XRF Analysis

The content of elements (Zn, Cu, Mn) in biomass before and after biosorption was determined by X-ray fluorescence. Before analysis, all samples were homogenized to powder form. Measurements were taken using portable XRF analyzer Niton XL3t (Thermo Scientific, Waltham, MA, USA) with high-performance semiconductor detector type. All samples were analyzed in three repeats (results of analyses were arithmetic mean, the relative standard deviation was <5%). 

### 4.2. ICP-OES Analysis 

Samples of plant materials (1.0 g) was digested with spectrally pure nitric acid 69% m/m (5 mL), (Suprapur, Merck, Kenilworth, NJ, USA) at the temperature 200 °C in a microwave digestion system Start D, Milestone (Sorisole, Italy) equipped with the advanced reaction sensors for temperature and pressure control. Parameters of the process were optimized to assure complete digestion of samples. After digestion, samples were diluted 10 times with ultrapure water (Millipore Simplicity) to perform multielemental ICP-OES analysis.

The concentration of elements in digested biomass was determined by ICP-OES spectrometer Vista MPX, Varian (Sydney, Australia), optimized and calibrated for multielemental analysis taking into account the effect of the acid matrix. The analyses were carried out in laboratory Accredited by Polish Centre of Accreditation (PCA) according to PN-EN ISO/IEC 17025:2005. Quality assurance of the test results was achieved by using Combined Quality Control Standard from Ultra Scientific, North Kingstown, RI, USA. All samples were analyzed in three repeats (results of analyses were arithmetic mean, the relative standard deviation was <5%).

## 5. Conclusions

The number of applications of biosorption process is growing and hence methods and tools for qualitative and quantitative description of the efficiency of the process should be developed. X-ray fluorescence characterized by good correlation with the widely used ICP-OES makes it an effective tool for the description of biosorption efficiency.

In the present paper, the results of these two analytical techniques were compared through cross-calibration and their advantages and drawbacks were emphasized. The simplicity, low cost of analysis and high accuracy of XRF make it an interesting method, alternative for more time- and materials-consuming techniques such as ICP-OES particularly useful for development of products and process. In conducted experiments it was shown that XRF can be used for the rapid screening tests in order to determine the content of main micronutrients on the biomass surface. Elaboration of cheap and quick analytical methodology is an important aspect in development of new processes and products based on biosorption process.

## Figures and Tables

**Table 1:** Twelve principles of GAC [2].

1. Direct analytical techniques should be applied to avoid sample treatment.	7. Generation of a large volume of analytical waste should be avoided and proper management of analytical waste should be provided.
2. Minimal sample size and minimal number of samples are goals.	8. Multi-analyte or multi-parameter methods are preferred versus methods using one analyte at a time.
3. In situ measurements should be performed.	9. The use of energy should be minimized.
4. Integration of analytical processes and operations saves energy and reduces the use of reagents.	10. Reagents obtained from renewable source should be preferred.
5. Automated and miniaturized methods should be selected.	11. Toxic reagents should be eliminated or replaced.
6. Derivatization should be avoided.	12. The safety of the operator should be increased.

**Table 2:** Zn, Cu and Mn content of post-extraction berries seeds residues determined by ICP–OES and XRF (mg/kg).

Sample	Zn		Cu		Mn	
	ICP-OES	XRF	ICP-OES	XRF	ICP-OES	XRF
Strawberry	43.6 ± 5.7	71.6 ± 3.9	13.5 ± 1.8	29.2 ± 1.0	85.8 ± 11.2	<LOD
Strawberry Cu	151 ± 20	36.9 ± 9.7	**9580 ± 1920**	**21,400 ± 4280**	25.6 ± 3.3	<LOD
Strawberry Zn	**5030 ± 1010**	**9570 ± 1910**	12.9 ± 1.7	76.4 ± 10.0	32.8 ± 4.3	<LOD
Strawberry Mn	59.1 ± 7.7	110.4 ± 8.0	87.3 ± 11.3	139 ± 14	**5130 ± 1030**	**11,000 ± 2200**
Blackcurrant	32.9 ± 4.3	52.3 ± 3.9	12.3 ± 1.6	26.5 ± 0.9	30.6 ± 6.1	<LOD
Blackcurrant Cu	241 ± 31	143 ± 6	**12,800 ± 2560**	**28,100 ± 5620**	29.8 ± 3.9	<LOD
Blackcurrant Zn	**10,800 ± 2160**	**26,800 ± 5360**	11.7 ± 1.5	87.7 ± 16.3	30.7 ± 4.0	<LOD
Blackcurrant Mn	18.2 ± 2.4	78.5 ± 4.9	36.6 ± 4.8	187 ± 23	**3210 ± 642**	**15,900 ± 3180**
Raspberry	34.6 ± 4.5	66.4 ± 3.6	8.96 ± 1.16	24.5 ± 1.5	75.9 ± 9.9	<LOD
Raspberry Cu	171 ± 22	< LOD	**12,600 ± 2520**	**27400 ± 5480**	14.0 ± 1.8	<LOD
Raspberry Zn	**4780 ± 956**	**8180 ± 1640**	9.09 ± 1.18	71.2 ± 11.7	18.8 ± 2.5	<LOD
Raspberry Mn	26.1 ± 3.4	138 ± 50	71.5 ± 9.3	404 ± 46	**2480 ± 496**	**11,800 ± 2360**

Bold—content in the enriched by biosorption biomass. <LOD—below limit of detection (30 mg/kg).

**Table 3:** Enrichment coefficients for post-extraction residues determined from ICP–OES and XRF.

Residue	*α*: ICP–OES			*α*: XRF		
	Zn	Cu	Mn	Zn	Cu	Mn
Strawberry	115	709	60	134	734	-
Blackcurrant	328	1037	105	512	1062	-
Raspberry	138	1407	33	123	1120	-

**Table 4:** Comparison of the coefficient (XRF/ICP-OES) of the microelement content in the biomass.

Post-Extraction Residue	Element	XRF/ICP–OES	XRF/ICP–OES
		Natural Biomass	Enriched Biomass
Strawberry	Zn	1.64	1.90
Blackcurrant		1.59	2.48
Raspberry		1.92	1.71
Strawberry	Cu	2.16	2.23
Blackcurrant		2.15	2.20
Raspberry		2.73	2.17
Strawberry	Mn	-	2.14
Blackcurrant		-	4.95
Raspberry		-	4.76

**Table 5:** Correlation equations for C_ICP-OES_= f(C_XRF_), mg/kg.

Element	Correlation Equation	Determination Coefficient
Cu	C_ICP-OES_ = 0.4564·C_XRF_ − 27.468	R^2^ = 0.9998
Zn	C_ICP-OES_ = 0.4193·C_XRF_ + 201.37	R^2^ = 0.9791
Mn	C_ICP-OES_ = 0.2594·C_XRF_ + 93.611	R^2^ = 0.8065

**Table 6:** Correlation equations for C_SEM-EDX_ = f(C_XRF_), mg/kg.

Element	Correlation Equation	Determination Coefficient
Cu	C_SEM-EDX_ = 0.0004·C_XRF_ + 3.5119	R^2^ = 0.5494
Zn	C_SEM-EDX_ = 0.0003·C_XRF_ + 4.5155	R^2^ = 0.4492
Mn	C_SEM-EDX_ = 0.0002·C_XRF_ + 0.7462	R^2^ = 0.3364
